# Job Demands, Emotional Exhaustion, and Job Performance Among Nurses: The Moderating Role of Transformational Leadership From Healthcare Top Management

**DOI:** 10.1155/jonm/8888713

**Published:** 2026-07-21

**Authors:** Geneviève Bilodeau, Leslie-Ann Boily, Jean-François Gagnon, Sarah-Geneviève Trépanier, Stéphanie Austin, Claude Fernet

**Affiliations:** ^1^ Département de Psychologie, Université du Québec à Montréal, Montréal, Canada, uqam.ca; ^2^ Département de Gestion Des Resources Humaines, Université du Québec à Trois-Rivières, Trois-Rivières, Canada, uqtr.ca; ^3^ Département de Psychologie, Université de Sherbrooke, Sherbrooke, Canada, usherbrooke.ca; ^4^ Infrastructure de Recherche en Prévention et Promotion de la Santé (IRPPS), Santé Québec Mauricie-et-Centre-du-Québec - Universitaire, Trois-Rivières, Canada

**Keywords:** emotional exhaustion, job demands–resources model, job performance, moderated mediation, nursing management, transformational leadership

## Abstract

**Background:**

Emotional exhaustion is a core component of burnout and a major concern in nursing, where high job demands are associated with lower job performance. The job demands–resources (JD‐R) model provides a framework to understand how work conditions affect nurses’ functioning, yet the role of leadership enacted by healthcare top management as a distal contextual resource remains underexplored.

**Objective:**

To examine the mediating role of emotional exhaustion in the relationship between job demands and in‐role job performance among nurses, and whether nurses’ perceptions of transformational leadership enacted by healthcare top management moderate this indirect effect.

**Methods:**

A cross‐sectional survey was conducted among nurses in the province of Quebec, Canada (*N* = 513). Structural equation modeling was used to test a moderated mediation model.

**Results:**

Job demands were positively associated with emotional exhaustion, which in turn relates negatively to job performance. Emotional exhaustion partially mediated the relationship between job demands and performance. Notably, the indirect effect was weaker among nurses who perceived high levels of transformational leadership from top management, suggesting that this structurally embedded contextual resource attenuates this relationship.

**Conclusion:**

The findings support the health‐impairment process of the JD‐R model and underscore the buffering role of transformational leadership in mitigating the adverse association between job demands and nurses’ performance through emotional exhaustion.

**Implications for Nursing Management:**

Healthcare organizations should prioritize leadership development at the top management level, as such leadership may contribute to a broader organizational climate, as perceived by nurses. These efforts may help reduce emotional exhaustion and sustain nurses’ in‐role performance in demanding care environments.

## 1. Introduction

Emotional exhaustion is widely recognized as the core component of burnout [1, 2]. It is particularly prevalent among healthcare professionals, especially nurses, who are frequently exposed to emotionally demanding situations and chronic stressors [3, 4]. Characterized by a prolonged state of emotional depletion and overextension, emotional exhaustion has been consistently associated with negative outcomes at both the individual level, such as reduced motivation and commitment, and the organizational level, including increased turnover intention and diminished job performance [5, 6].

To better understand the mechanisms underlying these outcomes, the job demands–resources (JD‐R; [7]) model offers a comprehensive framework. According to this model, unfavorable job characteristics contribute to emotional exhaustion through a health‐impairment process, whereby excessive job demands gradually erode employees’ energy and impair functioning [7]. Although this process has received substantial empirical support, it remains less explicit regarding the conditions under which such erosion may be attenuated. In high‐pressure environments such as healthcare, where emotional exhaustion is pervasive, the presence of contextual resources such as transformational leadership may be associated with differences in how this process is experienced [8]. Specifically, leadership enacted by healthcare top management, when perceived positively, may be reflected in nurses’ global perceptions of their work environment, thereby influencing how they experience and respond to demanding conditions [9].

Transformational leadership involves behaviors that communicate a clear and inspiring vision, foster trust, and encourage employees to pursue collective goals beyond their immediate self‐interest [10]. When enacted by top management and perceived by nurses, such leadership may operate through strategic and symbolic processes that shape a broader organizational context supporting employees in coping with job demands [11]. In this sense, it reflects a distal, structurally embedded resource rather than a proximal supervisory influence, with implications for employee well‐being and performance in complex healthcare environments.

**FIGURE 1:**
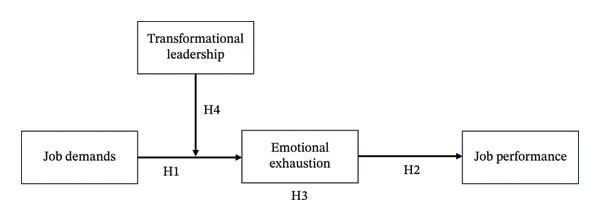
The proposed moderated mediation model. Note. H1–H4 represent the study hypotheses.

Accordingly, the present study proposes a moderated mediation model in which job demands negatively predict nurses’ job performance through emotional exhaustion. This indirect effect is expected to be attenuated when nurses perceive high levels of transformational leadership from healthcare top management (see Figure 1). By integrating leadership into the JD‐R framework and considering its perceived enactment by institutional actors as a key contextual resource, the study advances our understanding of how structural and relational dynamics, as experienced by employees, interact with job demands, relate to psychological resources, and ultimately shape their performance in high‐pressure healthcare environments.

The contribution of this research unfolds in three important ways. First, it sheds light on the relationship between job demands and positive outcomes such as in‐role job performance. While the JD‐R model posits that these outcomes are primarily driven by the presence of job resources, a proposition supported by substantial empirical evidence [7], prior and more recent studies suggest that job demands may also negatively affect performance through the mediating role of emotional exhaustion. For example, job demands have been found to be negatively associated with in‐role performance via higher levels of emotional exhaustion, whereas job resources are associated with extra‐role performance through higher work engagement (e.g., [12]). More recent research further highlights that emotional exhaustion plays a central role in influencing how employees respond to their work environment, with implications that extend beyond the health‐impairment process to a broad range of organizational outcomes (e.g., [7]). These findings underscore the importance of emotional exhaustion in determining core job behaviors, particularly in demanding contexts such as healthcare.

Second, the study introduces transformational leadership from healthcare top management as a perceived, distal contextual resource that can buffer the negative indirect relationship between job demands and performance (via emotional exhaustion). While leadership has been considered a job resource in prior JD‐R‐based research [13], its moderating role in the health‐impairment pathway when enacted by institutional actors rather than immediate supervisors remains underexplored. By focusing on leadership from top management and as perceived by nurses, the study highlights a structural and relational boundary condition associated with lower psychological resource depletion and, in turn, higher job performance.

Third, the research contributes to a more context‐sensitive application of the JD‐R model by emphasizing how broader organizational factors and employee perceptions interact with job demands in relation to employee functioning. This perspective aligns with recent theoretical developments that advocate for expanding the JD‐R framework to include structural and relational dimensions [14], thereby enriching its explanatory power in complex work settings such as healthcare.

## 2. Theoretical Background

### 2.1. The JD‐R Model

The JD‐R model [7] serves as a comprehensive framework for understanding how job characteristics relate to employee well‐being and performance across occupations. It distinguishes between job demands, defined as physical, psychological, social, or organizational aspects of the job that require sustained effort and are therefore associated with physiological and psychological costs, and job resources, which help achieve work goals, reduce demands, or foster personal growth and development [15]. In nursing, these job demands include role‐related challenges (e.g., role overload, ambiguity, and conflict), emotional strain (e.g., managing difficult patients and ethically challenging care situations often linked to moral distress), cognitive load (e.g., solving complex problems, frequent interruptions, and time pressure), and physical demands (e.g., prolonged standing and patient handling). Job resources, on the other hand, may be cognitive, emotional, or physical, such as task discretion, peer support, or ergonomic tools, all of which contribute to a more sustainable and enriching work experience [7, 16, 17].

The JD‐R model posits two core psychological processes. The health‐impairment process explains how excessive job demands deplete employees’ energy, leading to strain reactions such as emotional exhaustion and reduced well‐being [7, 15]. The motivational process, on the other hand, describes how job resources foster motivation and adaptive outcomes, including work engagement and performance [15]. Although the JD‐R model has received substantial empirical support, several studies suggest that both job demands and resources may be involved in both processes, with emotional exhaustion playing a mediating role [12, 14, 18]. Emotional exhaustion appears particularly detrimental to in‐role performance, which refers to the fulfilling of formal job responsibilities [19]. Research indicates that surface acting, a coping strategy often triggered by emotional exhaustion, is associated with lower in‐role performance, partly due to the regulatory effort it requires [20]. As a form of emotion regulation, it may come at the cost of internal psychological resources, thereby contributing to diminished functioning [21, 22]. This pattern is consistent with the idea that emotional depletion is linked to difficulties sustaining core responsibilities [23], especially in demanding work environments.

Interestingly, one of the central propositions of the JD‐R model [7] is that emotional exhaustion results from an imbalance between job demands and resources. When resources are insufficient to meet the demands of the job, employees tend to report higher levels of strain. Certain contextual resources may help attenuate these associations. For example, Xanthopoulou et al. [24] found that job resources moderate the association between job demands and burnout, including emotional exhaustion and cynicism. This pattern has also been observed in studies showing that resources such as autonomy, feedback, and social support are associated with weaker relationships between demands and burnout [25]. More recent research confirms that adequate resources, particularly autonomy and social support, are linked to lower burnout and turnover intentions among nurses [17].

Within the health‐impairment process of the JD‐R model, this buffering effect supports the rationale for a moderated mediation mechanism. In this framework, job demands are expected to be negatively associated with job performance through emotional exhaustion, particularly when employees report limited contextual support to manage these demands. Among the contextual resources identified in organizational research, transformational leadership has emerged as a key factor associated with employee adaptation and well‐being [26]. Transformational leadership refers to behaviors that articulate a compelling vision, foster trust, stimulate innovation, and provide individualized support across the organization [27, 28]. In healthcare settings, leadership enacted by top management has been positively associated with nurses’ psychological empowerment, work engagement, and job performance, particularly in high‐pressure environments such as hospitals and intensive care units [11, 29]. Importantly, this form of leadership operates at a more distal and structural level than supervisory leadership, shaping shared perceptions of organizational priorities, support, and working conditions across units [30]. As such, it may influence the health‐impairment pathway through system‐level signals that affect how demands are appraised, rather than through direct task‐related interactions.

Systematic reviews in nursing consistently show that transformational leadership, including at senior and executive levels, is associated with higher levels of job satisfaction and organizational commitment among nursing staff, which in turn support performance and retention (e.g., [31, 32]). Beyond these outcomes, transformational leadership has also been linked to differences in how nurses interpret and respond to job demands. By fostering a supportive climate, such leadership is associated with more positive interpretations of job demands, as well as with lower emotional exhaustion. Empirical studies further indicate that transformational leadership functions as a critical job resource in nursing, being associated with lower levels of burnout and related strain reactions [33].

Despite growing interest in the health‐impairment pathway of the JD‐R model, limited attention has been paid to how the perceived presence of transformational leadership from top management may influence the relationship between job demands, emotional exhaustion, and performance. Although transformational leadership is generally associated with positive employee outcomes, its role as a contextual resource may depend on how it is perceived by employees. Accordingly, the strength of the associations between job demands, emotional exhaustion, and performance may vary as a function of perceived leadership support. In healthcare organizations, top management typically operates at a strategic level, often distant from the daily realities of frontline staff. Drawing on the behavioral plasticity theory [34], employees may differ in their sensitivity to contextual cues such as leadership, particularly under high levels of job demands and psychological strain. Under such conditions, organizational and social signals are likely to become more salient, influencing how individuals experience and respond to their work environment. In this context, leadership from top management that provides clear direction, fosters trust, and offers meaningful support may be associated with weaker links between job demands and both emotional exhaustion and performance. Conversely, when leadership is perceived as weaker, job demands are more likely to translate into emotional strain and diminished performance. This suggests that transformational leadership from top management may function as a distal boundary condition within the health‐impairment process, rather than as a direct source of support, by shaping how job demands are experienced by employees who are more vulnerable to strain.

Building on the health‐impairment process of the JD‐R model, the present study tests a moderated mediation framework linking job demands, emotional exhaustion, and in‐role job performance. Specifically, we first examine whether emotional exhaustion mediates the association between job demands and job in‐role performance. We then test whether this indirect association varies as a function of nurses’ perceptions of transformational leadership enacted by healthcare top management. Accordingly, we propose the following hypotheses:

 Hypothesis 1 (H1): Job demands are positively associated with emotional exhaustion.
 Hypothesis 2 (H2): Emotional exhaustion is negatively associated with job performance.
 Hypothesis 3 (H3): Job demands are indirectly associated with job performance through emotional exhaustion.
 Hypothesis 4 (H4): The indirect association between job demands and job performance through emotional exhaustion varies as a function of nurses’ perceptions of transformational leadership from healthcare top management, such that the association is weaker when perceived leadership is high.

## 3. Method

### 3.1. Design and Sample

This cross‐sectional study is part of a broader research program examining the health and well‐being of nurses in the context of professional integration in Québec, Canada. Eligible participants were registered nurses employed in healthcare settings in Québec at the time of data collection in 2010, with 5 years or less of professional experience. Participants were recruited using a convenience sampling approach. Nurses invited to participate by email were members of the Quebec Professional Nursing Association. The invitation described the objectives of the study, emphasized that participation was voluntary and confidential, and included a link to the online questionnaire. A total of 513 nurses completed the questionnaire (17.3% response rate). All participants provided informed consent, and the study received ethical approval from the institutional review board of the institution affiliated with the corresponding author.

Most participants were women (87.6%), with a mean age of 29.2 (SD = 3.5) and an average of 3.5 years of professional experience (SD = 3.5). Participants reported working daytime shifts (24.1%), evening shifts (28.6%), nighttime shifts (21.4%), or on a flexible schedule (25.9%). The majority held full‐time positions (56%) and were employed on a permanent basis (78.2%).

### 3.2. Measures

All instruments used in this study were administered in French. The validity and reliability of these measures have been supported in previous research [11, 35, 36]. Descriptive statistics and intercorrelations among study variables are presented in Table 1.

**TABLE 1:** Mean, standard deviation, and correlations among variables.

Variable	Scale	*M*	SD	1	2	3	4
1. Job demands	1–7	5.03	0.98	—			
2. Transformational leadership	1–5	2.77	1.00	−0.283^∗∗^	—		
3. Emotional exhaustion	0–6	2.58	1.32	0.471^∗∗^	−0.287^∗∗^	—	
4. Job performance	1–7	6.14	0.71	0.040	0.085	−0.115^∗∗^	—

^∗∗^ *p* < 0.01.

### 3.3. Job Demands

Emotional, cognitive, and physical job demands were assessed using the Demand‐Induced Strain Compensation (DISC 2.0) Questionnaire [37]. Consistent with prior JD‐R research examining multiple job demands simultaneously, emotional, cognitive, and physical demands were modeled as indicators of a higher‐order latent job demands construct (e.g., [35]). Participants indicated how frequently they experienced each statement on a 7‐point scale ranging from 1 (*never*) to 7 (*almost always*). Sample items include the following: “I have to deal with people whose problems affect me emotionally” (emotional demands; 4 items; *α* = 0.77), “I have to display high levels of concentration and precision at work” (cognitive demands; 4 items; *α* = 0.85), and “I have to perform a lot of physically strenuous tasks to carry out my job” (physical demands; 4 items; *α* = 0.88).

### 3.4. Transformational Leadership

Perceptions of transformational leadership from top management were assessed using the Global Transformational Leadership (GTL) Scale [38]. Participants rated each item on a 5‐point scale ranging from 1 (*never*) to 5 (*frequently, almost always*). A sample item is “Encourages us and recognizes our work” (7 items; *α* = 0.94).

### 3.5. Emotional Exhaustion

Emotional exhaustion was assessed using the corresponding subscale of the Maslach Burnout Inventory–General Survey (MBI‐GS; [39]). Items were rated on a 7‐point scale from 0 (*never*) to 6 (*every day*). A sample item is “I feel used up at the end of a workday” (5 items; *α* = 0.90).

### 3.6. Job Performance

In‐role job performance was measured using the scale developed by Williams and Anderson [40]. Participants responded on a 7‐point scale ranging from 1 (*strongly disagree*) to 7 (*strongly agree*). A sample item is “I adequately complete the tasks that are assigned to me” (4 items; *α* = 0.90).

### 3.7. Analysis

Structural equation modeling (SEM) was used to test the hypothesized mediation model, as well as the moderated mediation model including latent interactions. Prior to conducting the main analyses, key statistical assumptions were assessed to ensure the validity and reliability of the results. The correlation matrix (Table 1) indicated sufficient variability among the variables, and no concerns regarding multicollinearity or singularity were observed. Analyses were conducted in two sequential steps. First, a mediation model was estimated to examine the indirect association between job demands and job performance through emotional exhaustion. Second, a moderated mediation model was tested by including transformational leadership from top management as a moderator of the association between job demands and emotional exhaustion. All models were estimated using Mplus Version 8.10 [41]. The robust maximum likelihood estimator (MLR) was used to account for potential violations of multivariate normality. All variables were centered prior to analysis to facilitate the interpretation of interaction effects. Significant interactions were probed using simple slope procedures described by Hayes [42], with high and low levels of transformational leadership defined as one standard deviation above and below the mean, respectively. Indirect effects were estimated using a bootstrap resampling procedure with 5000 iterations and a 95% confidence interval. Missing data were handled using the full information maximum likelihood (FIML) method, as recommended by Enders [43]. The sample size was considered adequate for the analytical approach and model complexity, given the use of SEM techniques and consistent with established guidelines for sample size in SEM (e.g., [44]).

## 4. Results

### 4.1. Direct Effects

The basic predictive model was just identified (df = 0) and showed perfect fit indices (CFI = 1.00; TLI = 1.00; RMSEA = 0.00). Results indicated that job demands were positively associated with emotional exhaustion (*β* = 0.471, *p* < 0.01), while emotional exhaustion was significantly and negatively related to job performance (*β* = −0.180, *p* < 0.01). These results support H1 and H2.

### 4.2. Indirect Effects

Within the basic predictive model, results revealed a significant indirect association between job demands and job performance through emotional exhaustion, along with a direct positive association between job demands and job performance (*β* = 0.126, *p* < 0.05). These findings indicate a pattern consistent with partial mediation, supporting H3.

### 4.3. Conditional Indirect Effects

**FIGURE 2:**
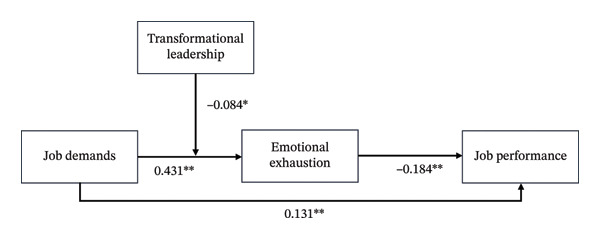
Structural model with standardized coefficients. Note: standardized coefficients (β) from the final structural equation model including latent interactions. ^∗^ *p* < 0.05; ^∗∗^ *p* < 0.01.

**FIGURE 3:**
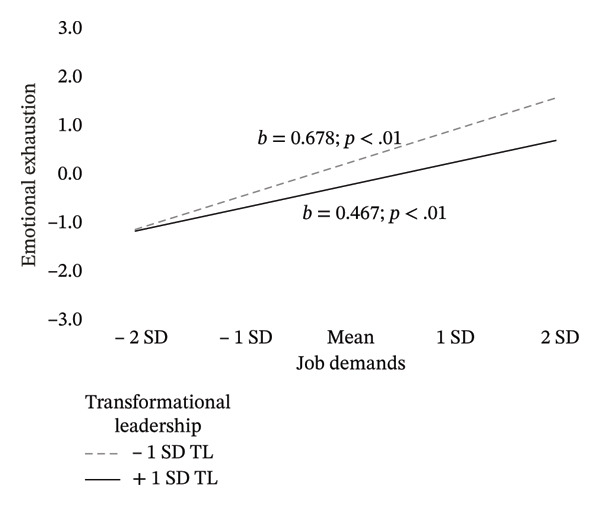
Simple slope of the effects of demands on emotional exhaustion as a function of transformational leadership from top management. Note: high and low levels of transformational leadership represent +1 and −1 standard deviations from the mean, respectively. TL = transformational leadership. Unstandardized regression coefficients are reported.

Results from the predictive model with latent interactions, for which conventional fit indices are not available with this estimation approach, showed that when transformational leadership from top management was included as a moderator, the associations between job demands and emotional exhaustion (*β* = 0.431, *p* < 0.01), between job demands and job performance (*β* = 0.131, *p* < 0.01), as well as between emotional exhaustion and job performance (*β* = −0.184, *p* < 0.01) remained significant (see Figure 2). The interaction between job demands and transformational leadership predicting emotional exhaustion (*β* = −0.084, *p* < 0.05; see Table 2) was also significant. Further analysis showed that the association between job demands and emotional exhaustion was weaker at high levels of transformational leadership (+1 SD; *b* = 0.467, *p* < 0.01) than at low levels of transformational leadership (−1 SD; *b* = 0.678, *p* < 0.01), supporting H4. The interaction is illustrated in Figure 3.

**TABLE 2:** Predictive results.

	Emotional exhaustion		Job performance		
	*b* (s.e.)	*β*	*b* (s.e.)	*β*	
*Basic predictive model*					
Job demands	0.628 (0.051)^∗∗^	0.471^∗∗^	0.090 (0.036)^∗^	0.126^∗^	
Emotional exhaustion			−0.097 (0.028)^∗∗^	−0.180^∗∗^	
*R* ^2^	0.221^∗∗^	0.027			

*Predictive model with latent interactions*					
Job demands (JD)	0.573 (0.054)^∗∗^	0.431^∗∗^	0.094 (0.036)^∗^	0.131^∗∗^	
Transf. leadership (TL)	−0.227 (0.056)^∗∗^	−0.173^∗∗^			
JD *x* TL	−0.106 (0.052)^∗^	−0.084^∗^			
Emotional exhaustion			−0.099 (0.028)^∗∗^	−0.184^∗∗^	
*R* ^2^		0.255^∗∗^	0.028^∗^		

	***a***		***b*** (s.e)_	**LLCI**	**ULCI**

*Job demands: single slopes*					
−1SD (Transf. leadership)	0.227		0.678 (0.083)	−0.111	−0.031
M (Transf. leadership)	0.000		0.573 (0.054)	−0.092	−0.027
1SD (Transf. leadership)	−0.227		0.467 (0.067)	−0.080	−0.021

*Note:* Transf. = transformational; *R* ^2^ = squared multiple correlation (reflecting the proportion of explained variance); *a* = regression intercept (used to draw the simple slope graphs); *b* = unstandardized coefficient; *β* = standardized coefficient; LLCI = lower limit confidence interval; ULCI = upper limit confidence interval.

Abbreviation: s.e. = standard error.

^∗^ *p* < 0.05.

^∗∗^ *p* < 0.01.

## 5. Discussion

Drawing on the health‐impairment process of the JD‐R model [7], this study examined whether transformational leadership from top management is associated with a weaker relationship between job demands and in‐role performance via emotional exhaustion. The results supported the hypothesized pathway, showing that job demands were positively associated with emotional exhaustion, which in turn negatively predicted in‐role job performance. Importantly, the findings suggest that the indirect association may be weaker among nurses who perceived higher levels of transformational leadership, pointing to key boundary conditions of this process. However, the indirect association remained significant even under high levels of perceived leadership, indicating that while leadership can play a meaningfully buffering role, it may not be sufficient on its own to fully counteract the energy‐depleting nature of job demands. Nevertheless, the attenuation observed suggests that transformational leadership plays a substantial buffering role in the health‐impairment process.

### 5.1. Theoretical Contributions

One central theoretical advancement of this study lies in its clarification of how emotional exhaustion mediates the relationship between job demands and in‐role performance. While the health‐impairment process of the JD‐R model was originally developed to explain the emergence of health‐related problems (e.g., [15]), our findings extend its relevance to behavioral outcomes, particularly in demanding healthcare contexts. For nurses, the accumulation of physical, emotional, and cognitive demands is associated with the depletion of emotional resources, thereby undermining their ability to perform core job tasks. These results provide further support for the conservation of resources (COR) theory [45], suggesting that job demands may be linked to loss cycles that impair performance. This study sheds light on the mechanisms through which job demands affect performance, reinforcing the central role of emotional exhaustion in determining how employees respond to their work environment. It complements prior research focused on the motivational pathways (i.e., performance facilitated by job resources) by showing that health‐impairment processes also impact essential in‐role functioning (e.g., [7, 12]).

This study adopts a contingency perspective that helps to clarify the conditions under which the health‐impairment process is more or less likely to occur. Specifically, transformational leadership from top management functions as a contextual buffer, such that the relationship between job demands and emotional exhaustion, and in turn, in‐role performance, is weaker under higher levels of perceived leadership. Importantly, this form of leadership differs from proximal supervisory leadership by operating at a more distal and structural level, shaping shared perceptions of organizational priorities and support across the work context. As such, leadership enacted by institutional actors reflects system‐level signals that structure how job demands are appraised, rather than directly regulating day‐to‐day task demands.

This perspective is consistent with research suggesting that leaders can influence the work environment of nurses [32, 33, 46, 45], whether by reframing stressors as challenges, fostering a sense of purpose, or supporting individual needs. These functions align with core dimensions of transformational leadership, including the articulation of a compelling vision (inspirational motivation), the provision of individualized support (individualized consideration), and the promotion of adaptive thinking (intellectual stimulation), all of which may shape how nurses interpret and respond to demanding work situations [10]. In this sense, transformational leadership may operate by influencing how job demands are managed, rather than solely by altering the demands themselves. Our theoretical propositions and empirical evidence complement existing research focused on individual‐level resources (e.g., [33]) by highlighting the role of structural and relational resources, such as leadership enacted by institutional actors, as boundary conditions under which the associations between job demands and employee functioning vary.

Drawing on the behavioral plasticity theory [34], our findings suggest that employees may differ in their responsiveness to contextual cues, particularly when exposed to high job demands. In this sense, transformational leadership may operate as a stabilizing resource under which the associations between job demands, emotional exhaustion, and performance differ. Conversely, when perceived leadership is lower, employees may be more susceptible to the impairing effects associated with job demands. This theoretical lens reinforces the idea that leadership functions not only as a resource within the JD‐R framework but also as a boundary condition within the health‐impairment process. It also invites future research to examine other leadership styles (e.g., authentic and servant) and sources (e.g., supervisors and peers) that may interact with job characteristics to shape employee well‐being and performance.

This work also aligns with emerging research emphasizing the importance of exploring how job demands interact with broader organizational dynamics. By focusing on nurses’ perceptions of leadership from top management, we move beyond the immediate work environment to consider how structural and relational dynamics influence employee functioning. This perspective reflects recent developments emphasizing the flexibility of the JD‐R model to incorporate contextual factors that reflect the complexity of modern work settings (e.g., [14]). Our findings offer a deeper understanding of how employees’ perceptions of institutional leadership can modulate the effects of job demands, reinforcing the importance of integrating relational and structural dimensions into models of occupational stress and performance. This context‐sensitive approach enhances the explanatory power of the JD‐R framework and opens new avenues for theory development in high‐pressure environments such as healthcare.

### 5.2. Limitations and Future Research

This study has several limitations. First, its cross‐sectional design limits causal inferences. Longitudinal research would be better suited to capture the dynamic nature of the health‐impairment process and to more rigorously examine the temporal ordering of the associations observed in this study. Although the data were collected at a specific point in time, the relationships examined are based on a well‐established theoretical framework and are unlikely to be substantially affected by the timing of data collection. Second, the use of self‐report measures may introduce common method bias [47]. Future studies should incorporate multiple data sources, particularly for assessing job performance. However, the observed pattern of results, including differential associations and conditional indirect effects, suggests that common method variance is unlikely to fully explain the relationships identified in this study. Moreover, although the observed effects were consistent with the proposed model, their magnitude suggests that additional contextual and individual factors should be considered in future research to further explain variability in nurses’ performance. In addition, the measure of job demands used in this study did not allow for a distinction between hindrance and challenge stressors. As suggested by Crawford et al. [48], these two types of demands may have distinct implications for strain, motivation, and performance. Future research should consider differentiating between these dimensions to better understand how leadership interacts with specific types of demands. Third, although the response rate (17%) falls within the range reported in prior research on healthcare professionals and nursing populations, where response rates are often variable and sometimes low (e.g., [49, 50]), it may limit the representativeness of the sample, especially given the use of a convenience sampling strategy. As noted in nursing research methodology, convenience samples are common but may introduce selection bias and limit generalizability [51]. Finally, the study was conducted in a single Canadian province; replication in other regions and countries would enhance the generalizability of findings, including among nurses with more varied professional experience.

### 5.3. Practical Implications

The findings offer actionable insight for healthcare organizations seeking to reduce emotional exhaustion and enhance nurses’ job performance. First, given the protective role of transformational leadership, organizations should invest in leadership development programs targeting top management, given that such leadership can shape shared perceptions of organizational priorities and support across the work environment. Prior research shows that transformational leadership can be cultivated through training in communication, feedback, and emotional intelligence [52, 53], and recent studies confirm its positive association with work engagement and performance (e.g., [11]). Experimental interventions further suggest that such leadership can be strengthened through targeted programs, yielding benefits such as improved job performance and job satisfaction [53] as well as adaptability to change [54].

Second, organizations should strive to reduce excessive job demands and provide tailored resources to help nurses cope with their work environment. Research has shown that aligning resources with the nature of the demands, whether emotional, physical, or cognitive, is associated with more effective management of strain and improved performance [55]. This principle is central to the JD‐R model, which emphasizes that resources not only foster motivation but also help attenuate the adverse effects of demands on burnout and engagement. Recent studies indicate that when nurses perceive adequate autonomy, social support, and team cohesion, the negative association between high demands and well‐being outcomes is attenuated, contributing to better psychological functioning and service quality [3, 56]. Examples of effective resources include social support [17] and opportunities for autonomy and participation in decision‐making [57].

## 6. Conclusion

This study supports the health‐impairment process among nurses, showing that job demands are indirectly negatively associated with job performance through emotional exhaustion. It also identifies transformational leadership from top management as a key organizational resource that can buffer this process. These findings underscore investing in leadership practices at the top management level to support resilience and performance in high‐demand healthcare settings.

## Funding

This study was supported by Fonds de Recherche du Québec‐Société et Culture (2024‐SE3‐332198).

## Conflicts of Interest

The authors declare no conflicts of interest.

## Data Availability

The data that support the findings of this study are available from the corresponding author upon reasonable request.
